# An enhanced two-dimensional hole gas (2DHG) C–H diamond with positive surface charge model for advanced normally-off MOSFET devices

**DOI:** 10.1038/s41598-022-05180-4

**Published:** 2022-03-10

**Authors:** Reem Alhasani, Taichi Yabe, Yutaro Iyama, Nobutaka Oi, Shoichiro Imanishi, Quang Ngoc Nguyen, Hiroshi Kawarada

**Affiliations:** 1 Department of Nano Science and NanoEngineering, School of Advanced Science and Engineering, Waseda University, Shinjuku, Tokyo 169-8555 Japan; 2 Kagami Memorial Research Institute for Materials Science and Technology, Waseda University, 2-8-26 Nishiwaseda, Shinjuku, Tokyo 169-0051 Japan; 3 Research Organization for Nano and Life Innovation, Waseda University, 513 Waseda-Tsurumaki, Shinjuku, Tokyo 169-0041 Japan; 4 Department of Communications and Computer Engineering, School of Fundamental Science and Engineering, Waseda University, Shinjuku, Tokyo 169-0051 Japan; 5 National Center of Nano Technology and Semiconductor, King Abdulaziz City for Science and Technology, Riyadh, 12354 Saudi Arabia

**Keywords:** Energy science and technology, Engineering, Materials science, Nanoscience and technology

## Abstract

Though the complementary power field effect transistors (FETs), e.g., metal–oxide–semiconductor-FETs (MOSFETs) based on wide bandgap materials, enable low switching losses and on-resistance, p-channel FETs are not feasible in any wide bandgap material other than diamond. In this paper, we propose the first work to investigate the impact of fixed positive surface charge density on achieving normally-off and controlling threshold voltage operation obtained on p-channel two-dimensional hole gas (2DHG) hydrogen-terminated (C-H) diamond FET using nitrogen doping in the diamond substrate. In general, a p-channel diamond MOSFET demonstrates the normally-on operation, but the normally-off operation is also a critical requirement of the feasible electronic power devices in terms of safety operation. The characteristics of the C–H diamond MOSFET have been analyzed with the two demonstrated charge sheet models using the two-dimensional Silvaco Atlas TCAD. It shows that the fixed-Fermi level in the bulk diamond is 1.7 eV (donor level) from the conduction band minimum.  However, the upward band bending has been obtained at Al_2_O_3_/SiO_2_/C-H diamond interface indicating the presence of inversion layer without gate voltage. The fixed negative charge model exhibits a strong inversion layer for normally-on FET operation, while the fixed positive charge model shows a weak inversion for normally-off operation.  The maximum current density of a fixed positive interface charge model of the Al_2_O_3_/C-H diamond device is − 290 mA/mm, which corresponds to that of expermental result of Al_2_O_3_/SiO_2_/C-H diamond − 305 mA/mm at a gate-source voltage of − 40 V. Also, the threshold voltage *V*_th_ is relatively high at *V*_th_ = − 3.5 V, i.e., the positive charge model can reproduce the normally-off operation. Moreover, we also demonstrate that the *V*_th_ and transconductance *g*_m _ correspond to those of the experimental work.

## Introduction

Diamond is the most valuable p-type wide bandgap seimiconductor, thanks to its distinctive properties compared with other semiconductor materials, e.g., silicon carbide (SiC), germanium (Ge), and gallium nitride (GaN). The wide bandgap of diamond (5.45 eV) enhances the device toughness in addition to a high carrier’s mobility at 4500 cm^2^/Vs and 3800 cm^2^/V s for electron and hole, respectively^1^, and a high thermal conductivity at 22 W/cm k as well^2^. These unique properties of the diamond make it a promising p-type semiconductor to be used as the substrate of electronic devices and the surface channel of  FETs. The sub-surface is a significant region due to its direct impact on the FET operation. For example, hydrogen terminated (C–H) diamond surfaces were invesitigated in terms of negative electron affinity and surface p-type conduction, then demonstrated to be suitable for electron device application with surface stability^3^. In the case of MOSFETs, the hydrogen termination can effectively induce the conductivity channel with the interface charge (fixed charge) in the electronic device surface. This characteristic makes C–H diamond with p-channel conduction an emerging research topic, which develops feasible high power/high-frequency devices, including high-power FET for different applications, e.g., the inverter systems^4^. The  positively charged hydrogen atoms of surface C-H dipoles  facilitate the adsorption process of the negative charged adsorbates, which are attracted at the diamond surface from the atmosphere^5^. The surface negative charge sheet induces the 2DHG layer which is located nearby the interface with a high hole density around 10^13^ cm^−2^ (10^20^ cm^−3^ near surface)^6–8^. In contrast, when the surface of the diamond is terminated by oxygen the surface conduction originated from  2DHG disappears. When the crystal structure ends, unsatisfied bonds, called “dangling bonds” appear and the surface energy increases^9^. As the high surface energy is not desired, this excess surface energy should be decreased by terminating dangling bonds with H atoms.  Then, the negative electron affinity of the diamond at − 1.3 eV appears after H-termination depending on C–H dipoles^10^. This distinguished property has a strong relationship with a chemisorbed species on the C-H diamond surface^11^.

Up to now, our research team has successfully reproduced the characteristic of C–H diamond FET using the 2-dimensional (2D) negative charge sheet model^3,4^ without relying on the 2D acceptor model^12^. Typically, these negatively charged sites scatter the centers for carrier (holes) transport near the C–H surface^4^. Also, the 2DHG layer can be obtained on the C–H diamond surface using negative interface charge sheet to establish the depletion mode, called normally-on. The C–H diamond MOSFET device usually has a normally-on operation in this context, as identified and analysed by device simulation^4,13^. However, the normally-off operation is required for the electronic power device to confirm the electric system protection from the perspective of safety and device feasibility. Kitabayashi et al.^14^ achieved a normally-off operation of the C–H diamond MOSFET with a partially oxidized channel under the gate. Using nitrogen ion implantation, the device exhibits satisfied normally-off operation depending on nitrogen concentration^15^. Saito et al.^16^ achieved the normally-off operation of high-voltage AlGaN/GaN high-electron mobility transistors (HEMTs) for power electronic applications to reduce 2DEG density. In addition, Liu et al.^17^ confirmed the normally-off device operation using HfO_2_-gate MOSFETs. Fei et al.^18^ fabricated the two kinds of diamond MOSFETs electronic device, with an oxidized Si terminated (C–Si) diamond channel. In the study, there are undoped and heavily boron-doped in the contact area (source/drain), and both of the MOSFET devices exhibited normally-off FET characteristics. However, there has not been any reported mechanism of normally-off result. Here, we propose a positive interface charge model for the normally-off operation of the C-H diamond FETs.

 Nitrogen is deep donor, the level of which is 1.7 eV from the conduction band minimum in diamond^19^. To achieve the enhancement mode, i.e., normally-off, we simulated the positive charge sheet inserted at the Al_2_O_3_/C-H diamond interface. We investigated this model for controlling threshold voltage (*V*_th_) to achieve the normally-off operation. The *V*_th_ without applying interface charge becomes almost zero. Also, we take the fixed positive charge as low as possible ($1 \times 10^{11}$ cm^−2^) and use nitrogen (donor) in the concentration of around 10^16^ cm^−3^ and boron (acceptor) in the concentration of around 10^15^cm^−3^. Typically, nitrogen coexists with boron in the same crystal because diamond cannot be doped by nitrogen only^20^. However, in this research work, the normally-off C–H diamond MOSFET has been investigated by a fixed Fermi level in the bulk and positive interface charge model. When nitrogent concentration is higher than boron concentration, Nitrogen atoms as donor with an activation energy of 1.7 eV fix the Fermi level at the same energy. This technique is used to obtain a largely negative value of *V*_th_ indicating the normally-off operation in the diamond MOSFET devices under specific conditions, e.g., fixed positive charge of SiO_2_ close to the surface and fixed Fermi level by deep donor level in the bulk.

## Results

### Analysis via experimental work

**Figure 1:**
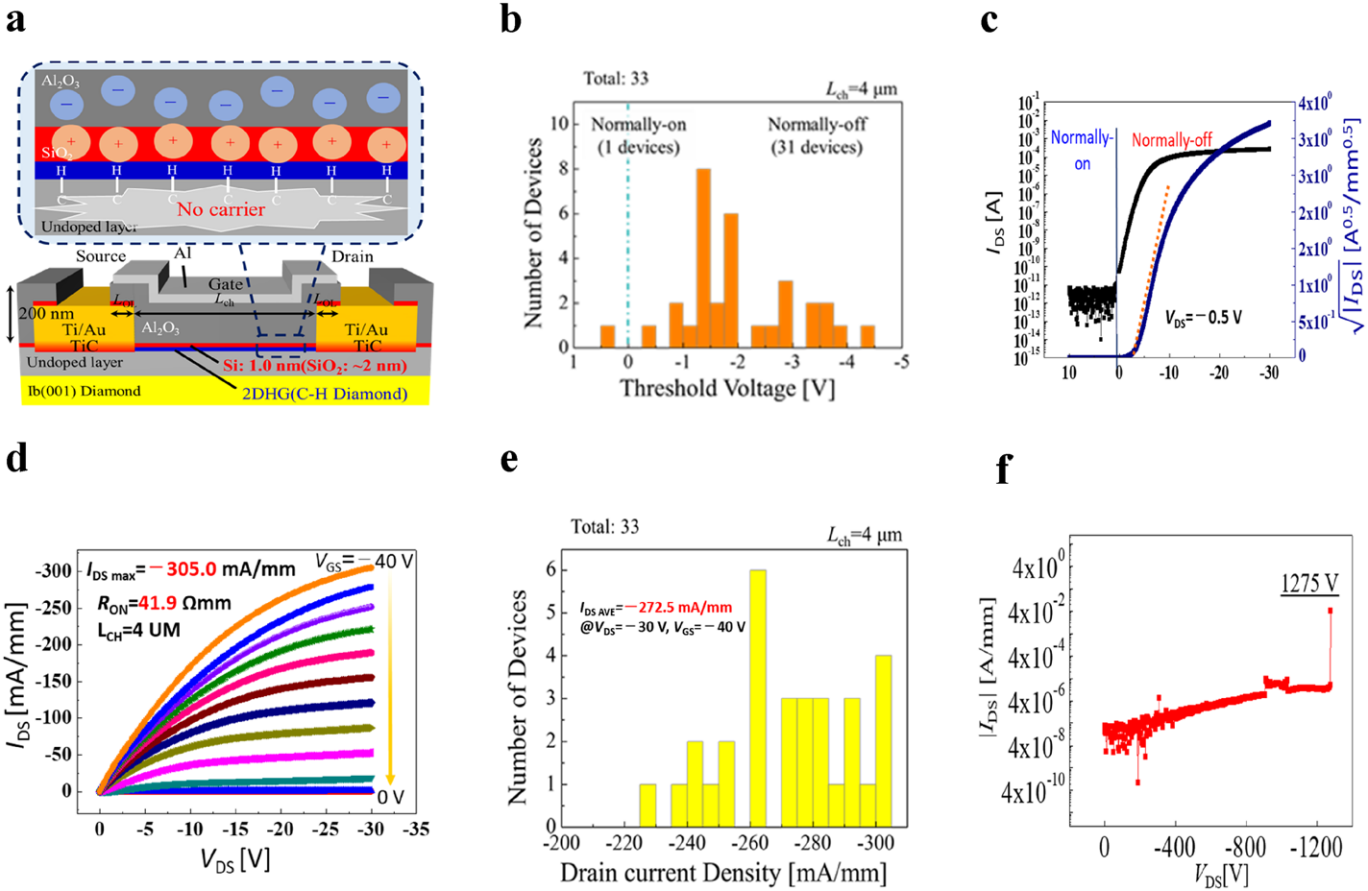
The *I*_DS_–*V*_*DS*_ characteristics of 2DHG Al_2_O_3_/SiO_2_/diamond MOSFET after confirming the SiO_2_ close to the surface, experimentally. **(a)** Cross-sectional representation of the 2DHG diamond MOSFET (blue line) with the SiO_2_ layer (red line) placed close to the interface. **(b)** *V*_th_ distribution of actual MOSFETs device with SiO_2_ of the 33 samples. The *V*_th_'s are within the range from 0.5 V to − 4.5 V, and almost all devices operate in the enhancement mode (normally-off). **(c)** *I*_DS_ −* V*_GS_ characteristic of SiO_2_/diamond MOSFET device, where the *V*_th_ = − 3.5 V at *V*_DS_ = − 0.5 V. **(d)** The diagram of *I*_DS_ − *V*_DS_ characteristics of SiO_2_/diamond MOSFET showed the maximum drain current obtained at *I*_DS MAX_ = − 305.0 mA/mm when drain voltage was fixed at − 30 V, * V*_GS_ was varied from − 40 V to 0 V with a voltage step of 4 V. **(e)** Drain current density distribution of actual MOSFET devices of 33 samples in which all devices achieved high drain current density (>220 mA/mm). **(f)** The breakdown voltage achieved at 1275 V with the gate drain distance *L*_GD_ = 20 μm.

In this work, the DC operation of the 2DHG diamond MOSFET (001) device is carried out via the fabrication of the 2DHG diamond MOSFET using SiO_2_ layer (2 nm) located under the gate for confirming normally-off operation (*V*_th_ $< 0$). Figure 1a shows the cross-sectional of MOSFET structure with a SiO_2_ layer, in which the source gate distance is *L*_SG_ = 2 μm, the gate length is *L*_G_ = 4 μm and the gate-drain distance is *L*_GD_ = 2 μm. We used the low boron (acceptor) concentration of $2 \times 10^{15}$ cm^−3^ and nitrogen (donor) concentration of 2 $\times 10^{16}$ cm^−3^ in the (001) substarte. The fundamental operation mechanism of this structure is that the SiO_2_ considered as a source of positive charge, prevents the holes from accumulating near the interface by the cancellation of negative charge at the interface, i.e., achieving normally-off operation by shifting *V*_th_ to negative value. The 2DHG is induced by the negatively charged sites of Al_2_O_3,_ except for the channel under the SiO_2_ layer, but it is reduced by positively charged SiO_2_ as mentioned. Figure 1b shows *V*_th_ distribution of the Al_2_O_3_/SiO_2_ diamond MOSFET device. The *V*_th_ range between 1 eV to − 5 eV at the device (32 devices) indicated that in almost all the cases, no normally-on operation was confirmed except for one device. The result confirmed that the normally-off operation was achieved in Al_2_O_3_/SiO_2_ diamond devices. The MOSFET device with a SiO_2_ layer exhibits the normally-off operation achieved at *V*_th_ = − 3.5 V that is suitable for power device application, as shown in Fig. 1c. The *V*_th_ value is determined as the value that decreases the drain current by 6 orders from the maximum drain current. The maximum drain current density is* I*_DS MAX_ = − 305.0 mA/mm at a drain voltage of *V*_DS_ = − 30 V and a gate voltage of *V*_GS_ = − 40 V, as illustrated in Fig. 1d. The drain current density distribution of actual MOSFET devices of 33 samples in which all devices achieved high drain current density, as shown in Fig. 1e. Also, the breakdown voltage was achieved in the Al_2_O_3_/SiO_2_ diamond MOSFET device with the gate-drain distance *L*_GD_= 20 μm  at 1275 V (Fig. 1f). Overall, we fabricated 2DHG Al_2_O_3_/SiO_2_ diamond MOSFETs and revealed that the normally-off operation can be obtained without deteriorating drain current density. This outcome is convenient for the analysis of MOSFETs operation by the device simulation using a fixed positive interface charge sheet model.

### Analysis via simulations

**Figure 2:**
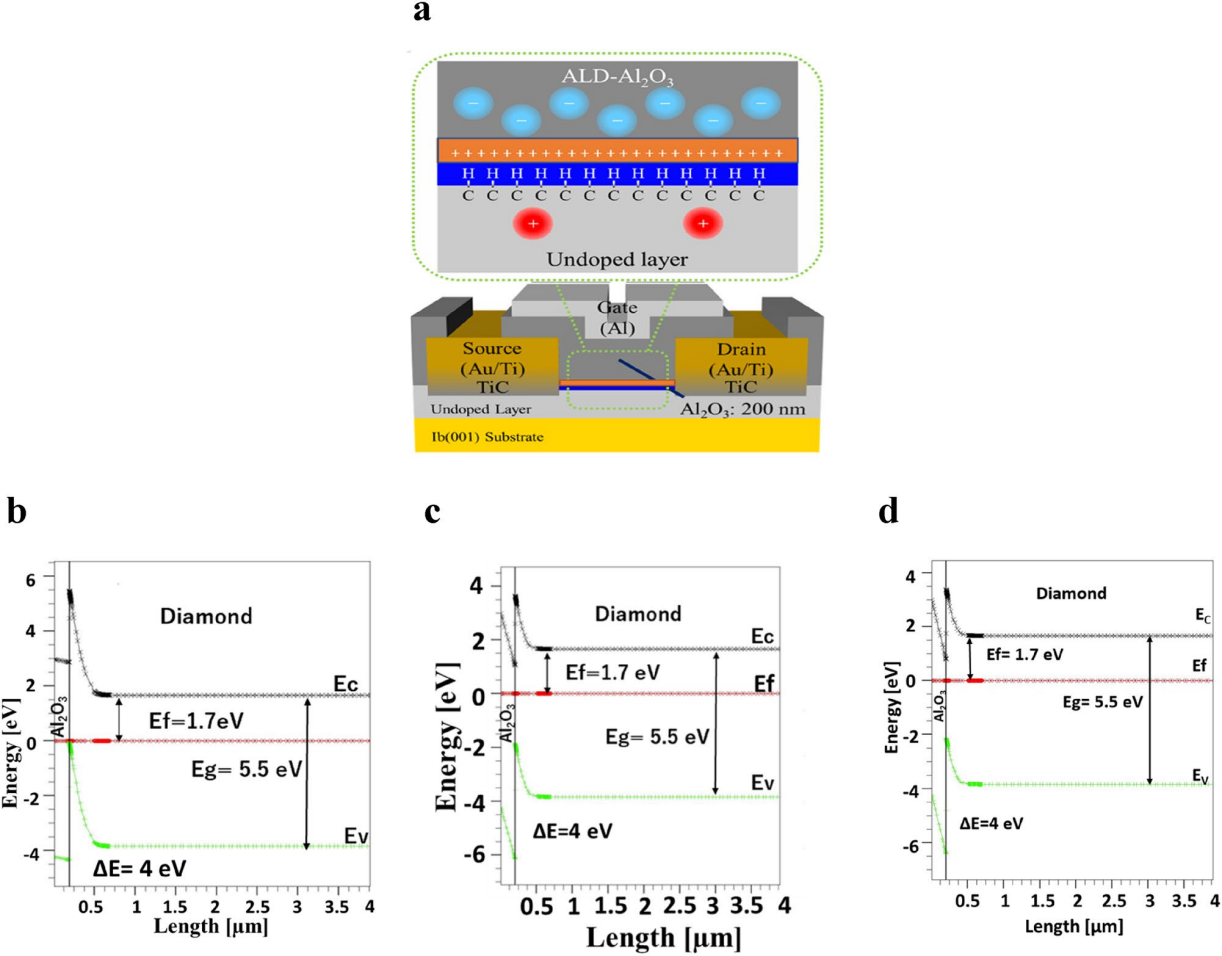
**(a)** Cross-sectional representation of the theoretical modeling 2DHG diamond MOSFET with the fixed interface charge sheet model close to surface in the FETs diamond substrate where *L*_GD_= 4 μm, *L*_ch_ = 4 μm and *W*_G_ = 25 μm. The upper section illustrated the negative interface charge in the ALD Al_2_O_3_.  **(b)**, **(c)**, **(d)** The band of the C–H diamond becomes upward near the Al_2_O_3_/C-H diamond interface. In the bulk, however, Fermi level *E*_f_ is fixed at 1.7 eV from the conduction band minimum corresponding to the position of nitrogen donor level  (1.7 eV), which are common in the following three models withe negative, neutral, and positive interface charge sheets. **(b)** The negative interface  charge density of 5 x 10^12^ cm^-2^. The valence band maximum crosses Fermi level near the Al_2_O_3_/diamond interface indicating a strong inversion layer. **(c)** For the no interface charge model, the valence band maximum is located at 1.8 eV from the Fermi level at the interface. It indicates a weak inversion layer. **(d)** The positive interface  charge density of 1 x 10^11^ cm^-2^. The valence band maximum forms an upward band bending,  but cannot reach the Fermi level. It is located at 2.1 eV from the Fermi level at the interface. The band diagram is also classified as a weak inversion.

The DC operation of 2DHG C–H diamond MOSFET device simulation is carried out via various interface charge sheet models. The normally-on operation is performed by the negative interface charge sheet model, whereas the positive interface charge sheet model with deep donor is dedicated to achieve the normally-off operation corresponding with experimental work illustrated in this paper. Also, the third model is a neutral interface charge sheet model that gives a possibility of controlling hole mobility due to no ion scattering, given that the ion scattering is described as a Coulomb interaction of the two particles. We considered the C–H diamond MOSFET, as depicted in Fig. 2a, with a gate length of *L*_G_ = 4 μm, a gate width of *W*_G_ = 25 μm, a passivation oxide ALD-Al_2_O_3_ with a thickness of *t*_ox_ = 200 nm, a source-drain distance (channel length) of *L*_SD_ = 4 μm, and a C–H diamond substrate with a thickness of 4 μm and the doping thickness of 4 μm.

#### Fixed Fermi level position and band diagram

The nitrogen doping with activation energy at 1.7 eV in low concentration in the diamond substrate (bulk) is a requirement to a fixed position of Fermi level close to the conduction band of p-channel C–H diamond. Also, using a positive fixed interface charge is another requirement to achieve the normally-off device operation.

We calculate the Fermi level position of the C–H diamond MOSFET with a low boron concentration of $2 \times 10^{15}$ cm^−3^ and a low nitrogen concentration of 2 $\times 10^{16}$ cm^−3^ in the freeze-out region. This concentration corresponds to the real condition of experiment research work conducted by our laboratory mentioned in this paper. Collins et al.^20^ showed that the donor in diamond is different from other materials, e.g., silicon or germanium. Typically, nitrogen in diamonds is hard to be ionized because ground level is exceptionally as deep as 1.7 eV. Then, it is regarded that the Fermi level could be clearly pinned at the level of 1.7 eV from the conduction band minimum by the effect of nitrogen doping (e.g. $10^{17}$cm^−3^) when nitrogen density is higher than that of boron as dopant (e.g. $10^{16}$ cm^−3^) in the same position. We apply the formula to calculate the Fermi position of carriers in the freeze-out region^20^ as follows:

1 $$E_{F} = \left( {E_{g} - E_{D} } \right) + K_{\beta } T ln\left( {\frac{{N_{d} - N_{a} }}{{2N_{a} }}} \right)\quad for\,N_{d} > N_{a}$$

where* E*_g_ is a bandgap, *E*_D_ is a donor ionization energy (activation energy), *K*_β_ is the Boltzmann constant, T is a temperature, *N*_d_ and *N*_a_ are the donor and acceptor concentration, respectively.

As shown in Fig. 2b, the Fermi level position is close to the valence band maximum near the interface between Al_2_O_3_ and C-H diamond.  In the bulk, however, deep nitrogen donor pins the Fermi level at about 1.7 eV from the conduction band minimum. It is calculated based on eq. (1). The reason behind Fermi level pinning in this position is that the electrons bounded by deep neutral donors, fix the Fermi level at 1.7 eV in the bulk^20^. The band diagram near the C–H diamond surface moves upward (upward band bending)until the valence band maximum crosses the Fermi level indicating  that high hole accumulation due to inversion layer is realized in the negative charge sheet model (Fig. 2b). From our prior study^4^, the 2DHG layer is confirmed when the fixed negative interface charge exists not only on the C–H diamond surface, but also in the passivation oxide layer Al_2_O_3_. It is usually formed by atomic layer deposition (ALD) on a C–H diamond surface. However, in the negative interface charge model, the bulk Fermi level position is pinned close to 1.7 eV from the conduction band minimum, because residual nitrogen concentration of 2 x 10^16^ cm^−3^  is higher than that of boron (2 x 10^15^ cm^-3^) as shown in Fig. 2b. Near the interface, the band diagram also turns upward in this case due to C–H diamond. The valence band maximum crosses the Fermi level at the interface calculated by the negative interface charge model, as shown in Fig. 2b.

Figure 2c also shows the band bending diagram with neutral interface fixed charge, where band bending is weakened compared with that of negative inteface charge model. At the interface, however, the valence band maximum is located at 1.8 eV from the Fermi level indicating the presence of a weak inversion layer.

**Figure 3:**
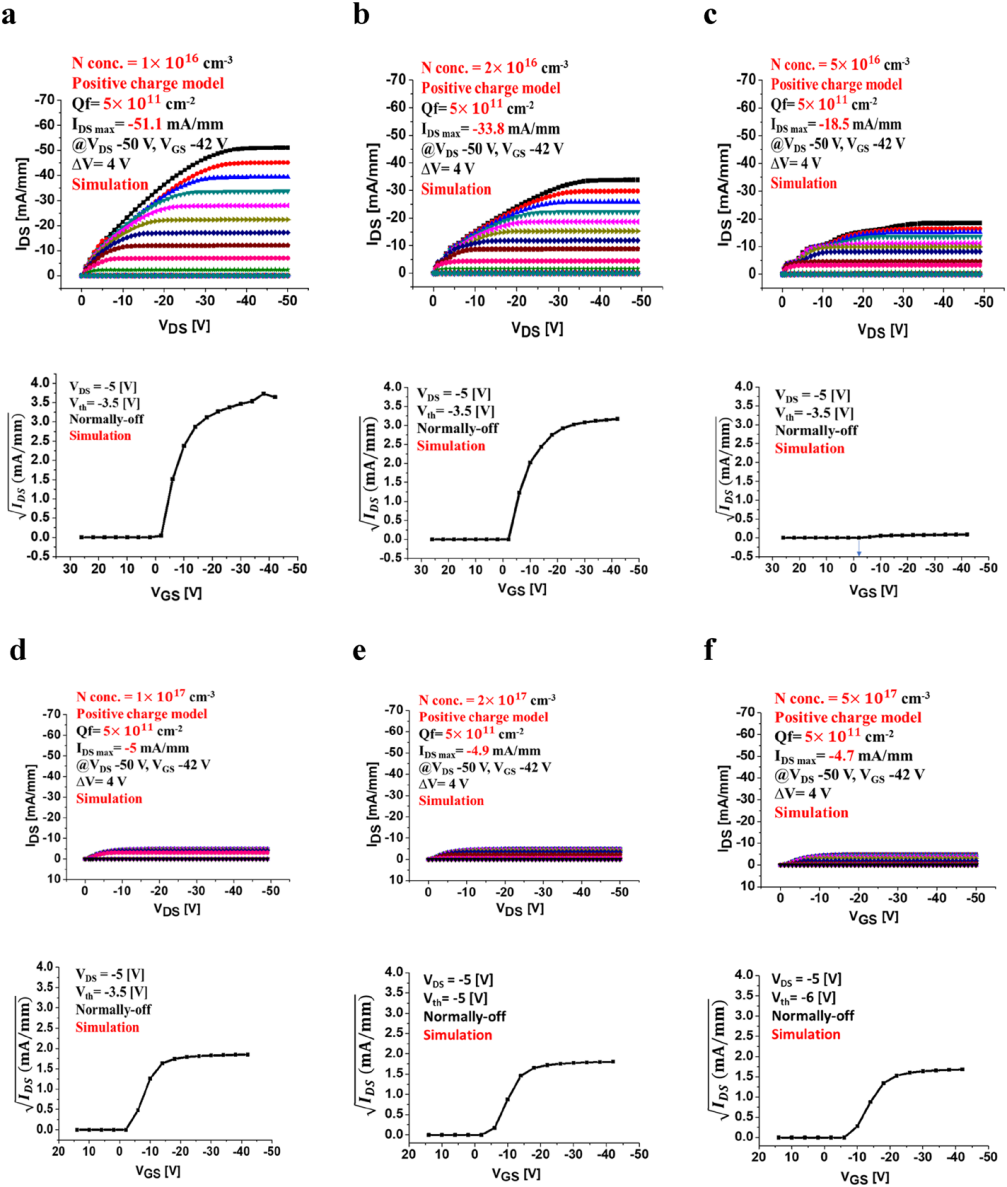
The theoretical modeling and output characteristics of 2DHG diamond MOSFET positive surface charge sheet model (simulation) of 5 × 10^11^ cm^-2^ with a boron doping concentration of 4 × 10^15^ cm^-3^ and various nitrogen doping layer at the room temperature. **(a)** The maximum drain current density is simulated at* I*_DS Max_ = − 52 mA/mm with nitrogen concentration of 1 × 10^16^ cm^-3^ and the *V*_th_ is at − 3 eV. **(b)** *I*_DS Max_ = − 33 mA/mm with nitrogen concentration of 2 × 10^16^ cm^-3^ and the *V*_th_ is at − 3 eV. **(c)** *I*_DS Max_ = − 19 mA/mm with nitrogen concentration of 5 × 10^16^ cm^-3^ and the *V*_th_ is at − 3 eV. **(d)*** I*_DS Max_ = − 5 mA/mm with nitrogen concentration of 1 × 10^17^ cm^-3^ and the *V*_th_ is at − 3 eV. **(e)** *I*_DS Max_ = − 5 mA/mm with nitrogen concentration of 2 × 10^17^ cm^-3^ and the *V*_th_ is at − 6 eV**. (f)** *I*_DS Max_ = − 2 mA/mm with nitrogen concentration of 5 × 10^17^ cm^-3^ and the *V*_th_ is at − 6 eV.

 A weak inversion layer still appears even when the positive interface charge sheet exists with the a real density of 1 x 10^11^cm^-2^. The valence band maximum is located 2.1 eV from the Fermi level. As a result the C-H diamond MOSFET with positive charge sheet becomes normally-off (enhancement mode), as shown in Fig. 2d. The reason is that the positive interface charge prevents the C–H surface from accumulating positive carriers (holes) to form  a channel. Hence, the electron potential at the C–H surface becomes low due to positive charge, which leads to a high barrier for hole that does not allow carrier flow from source to drain without applying a negative gate voltage. This indicates the normally-off operation. We then find out that the Fermi level position does not change with the different nitrogen concentrations as long as it exceeds that of boron. Hence, in this work, we focused on the nitrogen concentration to keep the Fermi level position which is allowed to shift *V*_th_ to a more negative value for achieving normally-off operation of the device. The negative shift of *V*_th_ is also obtained by hole recombination with electron of non-activated donor (neutral donor). When the holes enter the subsurface channel region doped with nitrogen (neutral donors), they can be recombined with electrons of  non-activated donors. After the recombination they become ionized donors which are positively charged. The presence of positive charge near the interface shift *V*_th_ more negative as mentioned above. By increasing the nitrogen concentration, the device needs to apply a higher voltage which leads to an increase in the shift of *V*_th_ threshold voltage to a more negative value for confirming the enhancement mode. A recent study demonstrated that the shift of *V*_th_ to a more negative value occurs because of an increase in nitrogen concentration corresponding to an increase in trap charge density due to a high nitrogen doping concentration in diamond^21^. Within the scope of this paper, we just show the simulation results of the affected nitrogen concentration of the positive surface charge case in this section. Figure 3a–f shows the FET simulation of various nitrogen concentrations in the diamond FETs with a positive surface charge model.

In the simulation theoretical modeling, we used the ideal Schottky contact with Schottky barrier height (SBH) of 0.1 eV between the source/drain metal (Au/Ti) and the C–H diamond surface of the MOSFET. In contrast, the large value of SBH cannot reproduce the *I*_DS_−*V*_DS_ and *I*_DS_−*V*_GS_ chracteristics^22^. Ohmic properties were obtained when metals with higher electronegativity, such as Pt, Au, Pd, and Ag, were used. In those cases, the SBHs of the diodes were assumed to be less than 0.3 eV^3^. Also, TiC was effective in formatting the Ohmic contact. The valence band offset $\left( {\Delta E_{V} } \right)$ between the edge of the valence band in C–H diamond and passivation layer Al_2_O_3_ is in the range of 3 ~ 4 eV.

#### Current–voltage characteristics

We then calculate the DC operation (*I*_DS_−*V*_DS_ characteristic) of the simulated MOSFET device by identifying the impact of drain-source current *I*_*DS*_ on the drain-source voltage *V*_*GS*_ in the linear scale of the positive fixed interface charge sheet model.

**Figure 4:**
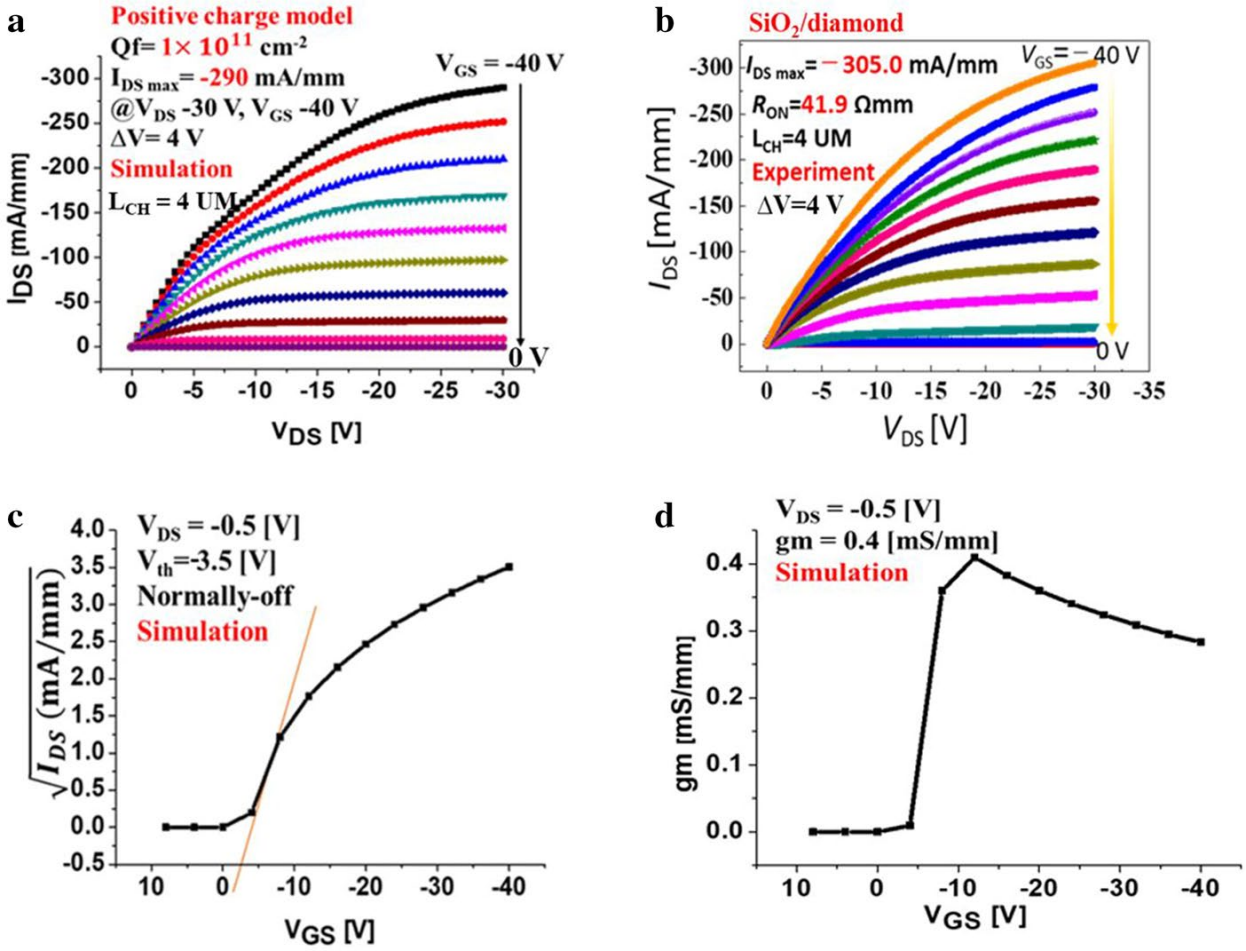
The simulated output characteristics of C−H diamond MOSFET positive interface charge model of 1 × 10^11^ cm^-2^ with boron doping concentration 2 × 10^15^ cm^-3^ and nitrogen doping layer in a concentration of 2 × 10^16^ cm^-3^ at room temperature. **(a)** The diagram of simulated *I*_*DS*_*−V*_*DS*_ characteristics, in which the drain density *I*_DS Max_ = − 290 mA/mm when *V*_DS_ is in the range of − 30 V, and *V*_G_ varies in the ranges from − 40 V to 8 V with a voltage step of 4 V, corresponding to the experimental work (b). **(b)** The diagram of experimented *I*_DS_−*V*_DS_ characteristics of SiO_2_/diamond MOSFET showed the maximum drain current obtained at *I*_DS MAX_=− 305.0 mA/mm when drain voltage was fixed at − 30 V, *V*_GS_ was varied from − 40 V to 0 V with a voltage step of 4 V. **(c)** The simulation of the *V*_th_ = − 3.5 V at drain voltage *V*_DS_ = − 0.5 V corresponds to (a) indicating the normally-off operation fitting with Fig. 1c. **(d)**. The plots of simulated results correspond to mobility varied at 100 cm^2^/Vs when transconductance of device g_m_ = 0.4 mS/mm.

The operation is completely pinch-off at a gate bias of 8 V and saturated in the Ohmic region when the applied negative gate bias is − 40 V, and the drain voltage is − 30 V, with a voltage step of 4 V. This theoretical result shows that we can achieve a high current density as a function of the drain voltage of the MOSFET with nitrogen-doped bulk when the gate bias is negative, i.e., *V*_GS_ < 0. Specifically, Fig. 4a illustrates the calculated output of *I*_DS_-*V*_DS_ characteristic with the positive fixed interface charge, in which maximum current density is* I*_DS Max_ = − 290 mA/mm when the channel length (distance between the source and drain) is 4 μm and overlapping gate length is 4 μm. This result is so similaer to expermental result that maximum current density is *I*_DS Max_ = − 305 mA/mm, Fig. 4b. The improvement of field-effect mobility is a requirement to confirm the high drain current density^23^. Also, the gate threshold voltage is *V*_th_ = − 3.5 V, as identified from the plot of *I*_DS_−*V*_GS_, i.e., the normally-off operation (enhancement mode) is achieved, as shown in Fig. 4c. Figure 4d shows that the transconductance *g*_m_ of the device is a constant of 0.4 mS/mm when the drain current *V*_DS_ is at − 0.5 V. The *V*_th_ value is controlled by the increased adsorption of the positive charge and/or the decreased adsorption of the negative charge. In addition, the main factor in achieving the normally-off operation is the application of the positive interface charge as a mechanism of surface charge effects on the channel conductivity. The deep donor doping in the substrate using nitrogen with 2 × 10^16^ cm^−3^ density was applied to fix the Fermi level position to obtain inversion channel. Figure 4b shows the *I*_DS_−*V*_GS_ characteristic, which reveals that the gate threshold voltage *V*_th_ leads to the fabrication of the enhancement mode device. This output result (Fig. 4a) of the simulated fixed positive interface charge model corresponds to the actual experimental work (Fig. 4b) using the SiO_2_ layer located at the interface between the gate insulator  Al_2_O_3_and the C−H diamond. In other devices, we also achieved a fit result of *V*_th_ and *I*_DS_−*V*_DS_ characteristics between simulated and experimetanl results.

**Figure 5:**
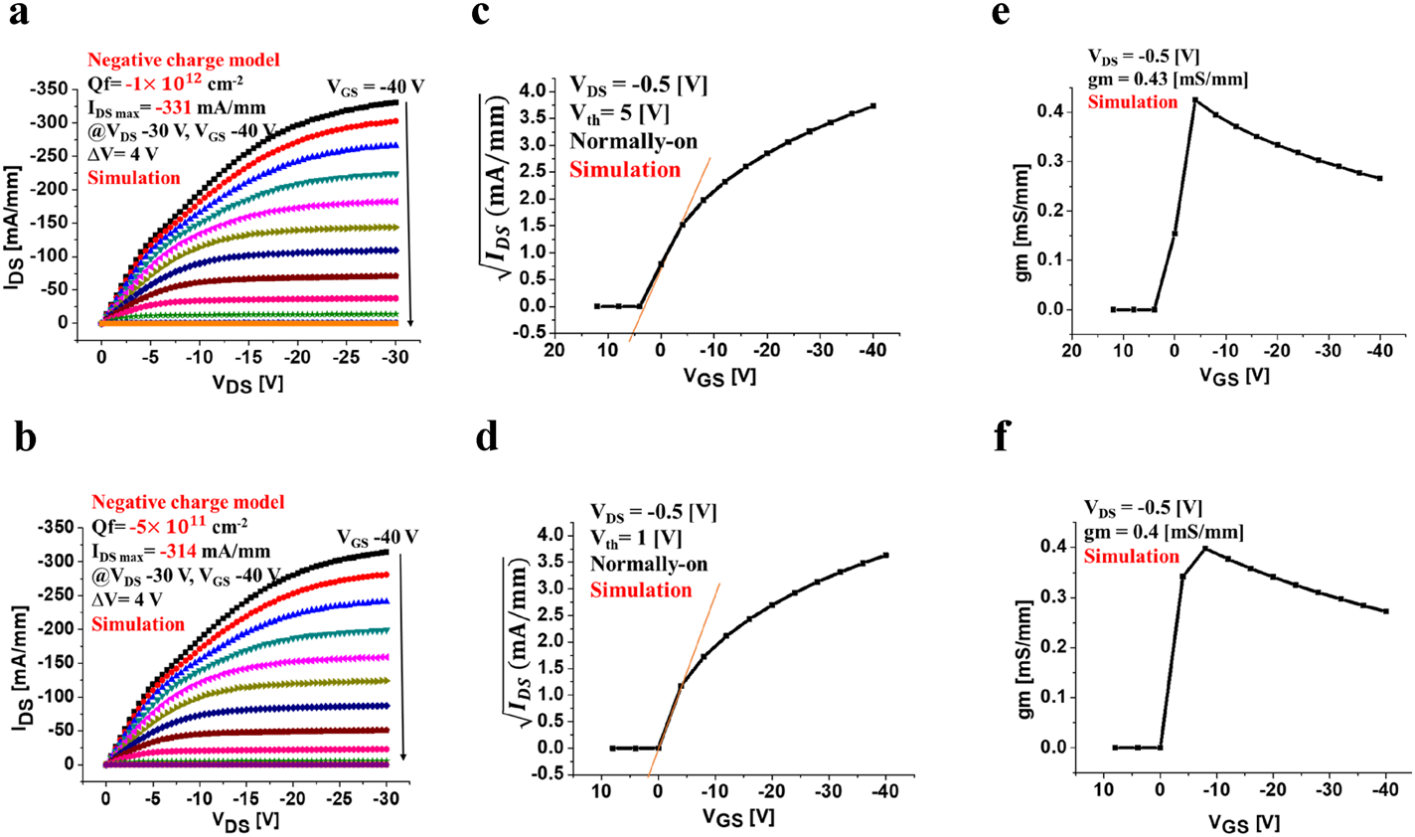
The simulated output characteristics of C–H diamond MOSFET in a negative interface charge model. **(a)** When applied negative interface charge at *Q*_f_ = −1 × 10^12^ cm^-2^ with nitrogen doping layer at 10^16^ cm^-3^, the maximum drain current density is simulated at *I*_DS Max_ = − 331 mA/mm with *V*_DS_ of − 30 V, and the gate voltage *V*_GS_ was varied in the ranges from − 40 V to 8 V with a voltage step of 4 V. **(b)** When applied surface charge at *Q*_f_ = − 5 × 10^11^ cm^-2^, the maximum drain current density is simulated at *I*_DS Max_ = − 314 mA/mm with *V*_DS_ of − 30 V, and the gate voltage *V*_GS_ was varied in the ranges from − 40 V to 8 V with a voltage step of 4 V. **(c)** Threshold voltage simulated at *V*_th_ = 5 V corresponding to (a) indicating the normally-on operation at *V*_DS_ = − 0.5 V. **(d)** Threshold voltage simulated at *V*_th_ = 1 V corresponding to (b) indicating the normally-on operation at *V*_DS_ = − 0.5 V. **(e)** The transconductance of device *g*_m_ = 0.43 mS/mm corresponding to (a). **(f)** The transconductance of device *g*_m_ = 0.4 mS/mm corresponding to (b).

**Figure 6:**
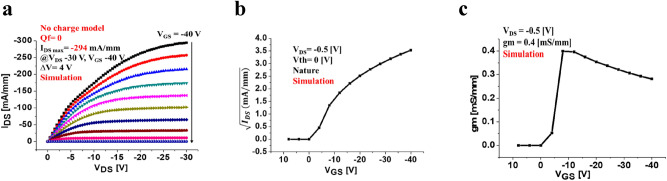
The simulated *I*_*DS*_*–V*_*DS*_ characteristics of 2DHG diamond MOSFET in the neutral surface charge model (non-charged). **(a)** The maximum drain current density is *I*_DSMax_ = − 294 mA/mm with *V*_DS_ of 30 V, and *V*_G_ ranges from − 40 V to 8 V with a voltage step of 4 V. **(b)** Threshold voltage simulated at *V*_th_ = 0 V indicating no operation of the device at *V*_DS_ = − 0.5 V. **(c)** The transconductance of device *g*_m_ = 0.4 mS/mm.

**Figure 7:**
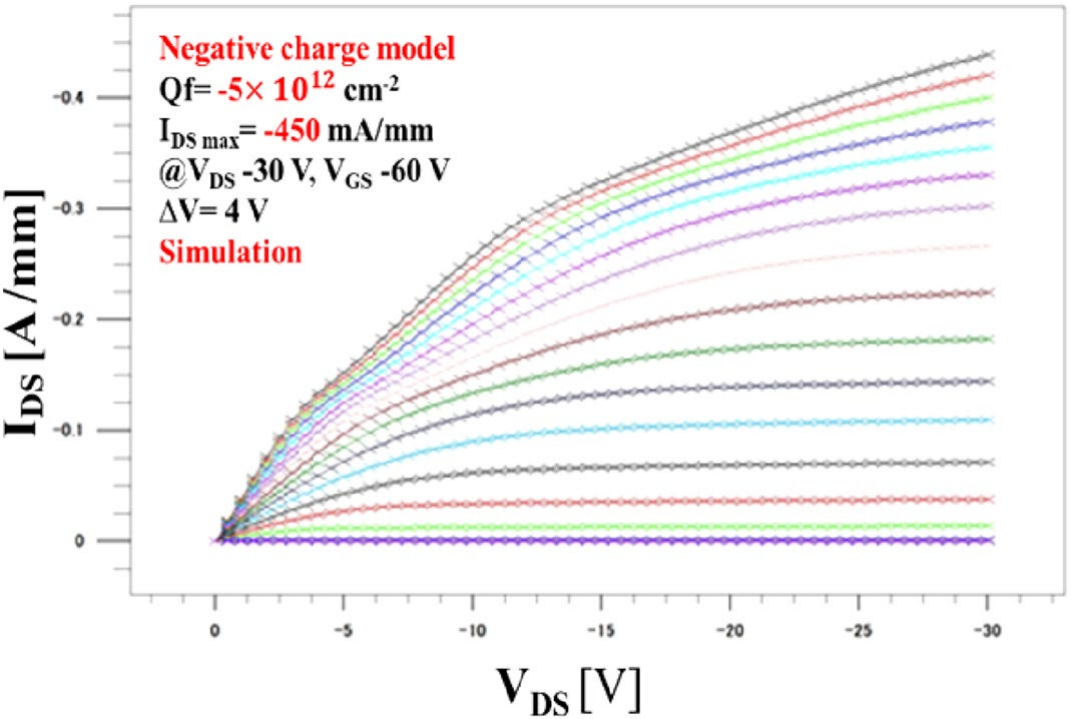
The theoretical modeling of *I*_*DS*_*–V*_*DS*_ characteristics of 2DHG diamond MOSFET with negative interface charge model at − 5 × 10^12^ cm^-2^. The drain current reaches its limitation with gate voltage range V_GS_ = − 60 V. It is caused by the saturation of  hole injection by the gate voltage.

As can be observed from the *I*_DS_−*V*_DS_ characteristic of the interface charge modeling using atlas TCAD device simulator in Fig. 5a,b, the drain current density *I*_DS_ of the C−H diamond MOSFET with the negative interface charge sheet model (− 1 × 10^12^ cm^−2^) and (− 5 × 10^11^ cm^−2^) exceeds − 331 mA/mm and − 314 mA/mm at a drain bias of − 30 V, respectively. The evaluation result also shows the saturation behavior in the ohmic region when the gate bias is greater than 0 V, and the pinch-off is observed when *V*_GS_ is 8 V. Figure 5c,d shows that the *V*_th_ is 5 V, 1 V at drain voltage − 0.5 V, which indicates the normally-on operation when the negative interface charge is *Q*_f_ = − 1 × 10^12^ cm^−2^ and *Q*_f_ = − 5 × 10^11^ cm^−2^, respectively. In addition, Fig. 5e,f shows that the transconductance of the device is a constant of 0.4 mS/mm when the drain current *V*_DS_ is at − 0.5 V. In the neutral charge model, the *I*_DS_-*V*_DS_ characteristic plot depicts the saturation behavior when the maximum drain current density is − 294 mA/mm, and the threshold voltage is already zero. Moreover, Fig. 6    shows the device simulated output characteristics* I*_DS_−*V*_DS_ and *V*_th_ in the neutral charge model, in Fig. 6a,b respectively. Figure 6c shows that the transconductance of the device is a constant of 0.4 mS/mm when the drain current *V*_DS_ is at − 0.5 V. In this case, no ion scattering in the interface leads to the possibility of a controlling carrier’s mobility. When hole mobility is increased in the device, we observe a sharp increase in drain current density saturation^13^. In this context, the C–H diamond MOSFET with a positive interface charge demonstrates the performance goal. The significant increase in *I*_DS_ is apparent compared to that of partial nitrogen ion implant MOSFET device when *L*_G_ and *L*_SD_ have the same values^14^. However, the drain current is still located in the saturation region, even when we increase the gate voltage to a very high value. The interface charge modeling evaluation shows that the drain current gets more linear behavior when we apply gate reverse bias till − 40 V, but when increasing the reverse bias beyond − 40 V until reaching saturation voltage, the current stops to increase. This means that the conductance reaches its limitation due to the limitation of  hole supply from the source contact which prevents the increase of drain current anymore, as shown in Fig. 7.

The decrease in the maximum drain current in the positive interface charge model (compared to that of the negative interface charge model) corresponds to the shifted value of the *V*_th_ to a negative value. Also, the main reason behind the decreasing drain current density of normally-off C-H diamond MOSFET is the high resistivity of the channel^14,15^.

Specifically, we determined that the 2DHG SiO_2_/diamond with transconductance of *g*_m_ = 0.89 mS/mm was obtained at a drain voltage of − 0.5 V, while the transconductance obtained at *g*_m_ = 0.4 mS/mm in the simulation with positive interface charge model. The simulation work then confirmed that the achieved normally-off operation using a positive interface charge model corresponds to the case of the MOSFET device that used the SiO_2_ layer and the obtained experimental results.

## Conclusion

In this research article, we simulate and discuss the characteristics of the MOSFET device with deep donor (*E*_D_ = 1.7 eV) nitrogen in a low and medium concentration (10^16^ and 10^17^ cm^-3^) in diamond substrate using the 2D drift–diffusion model. The experimental maximum drain current density is − 305 mA/mm. The simulation achieves similar result at − 290 mA/mm. Those values are the highest in normally-off diamond FET with a complete pinch-off and a saturation region. However, this value is still lower than the current in the case of a negative interface charge model in the saturation region in which high gate voltage causes the highest gate-drain resistivity. The gate threshold voltage can be controlled and shifted to the negative value of* V*_th_ = − 3.5 V when the applied positive interface charge is close to the interface, i.e., the normally-off operation (enhancement mode) is achieved. The obtained simulated results correlate with the experimental work using the SiO_2_ layer located between Al_2_O_3_ and C−H diamond. We then show how the surface band can be controlled when the substrate nitrogen doping was applied together with the selection of the interface charge. Also, the saturation behavior of the current in this model would be improved when the source resistance is reduced using other related techniques, e.g., p^+^ type doping in the contact area. These promising results then bring new insight into this research theme and demonstrate that the proposal can facilitate various applications of p-channel diamond MOSFET devices, e.g., complementary power MOSFETs with trench gates as vertical FETs or the smart inverter systems with bulk conductions, to enable high breakdown voltage and low on-resistance, with less switching loss.

## Methods

The device DC operation and gate threshold voltage *V*_th_ together with the *I*_DS_−*V*_DS_ characteristics of the C–H diamond MOSFET are evaluated by simulation using Atlas TCAD as the two-dimensional (2D) device simulator software (ATLAS User’s manual, *version 5.24.1.R.* et al. 2017, https://www.silvaco.com/))^24^. We used this software simulator to achieve both normally-off and normally-on characteristics by considering three typical C–H diamond MOSFET devices using various fixed interface charge models: negative interface charge, neutral-surface charge, and positive interface charge.

The device is under the thermal equilibrium conditions at 300 K. Figure 2 shows the C–H diamond MOSFET structure device modeling in an incomplete ionization model. The key parameters that are used for modeling devices include a diamond substrate with a thickness of 4 µm and Al gate length (*L*_G_) of 4 µm formed as the overlapping gate with a thickness of 100 nm. Also, Al_2_O_3_ is formed as ALD with a thickness of 200 nm, channel length (*L*_SD_) is 4 µm, source and drain contacts are formed using Au/Ti. Other key diamond material parameters are summarized in Table 1 in which we show the electron affinity of the diamond is − 1.3 eV.  We also assumed the incomplete ionization of impurities model in the freeze-out region, given that nitrogen shows an insulating behavior in the diamond. We then perform nitrogen and boron doping in diamond (4 µm) in the concentration of 2 × 10^16^ cm^−3^ and 2 × 10^15^ cm^−3^, respectively. Also, the 2DHG diamond MOSFET (001) is carried out via the fabrication of the 2DHG diamond MOSFET using SiO_2_ layer (2 nm) under the gate to confirm normally-off operation, in which the source gate distance is *L*_SG_ = 2 μm, the gate length is *L*_G_ = 4 μm and the gate-drain distance is *L*_GD_ = 2 μm. We used the values of boron and nitrogen concentration measured in the experiment work.

**Table 1:** Key parameters of the diamond material used for the modeling MOSFET device.

Parameters	Values of diamond
Bandgap E_g_	5.5 eV
Effective conduction band density of state (nc300)	9.4 × 10^18^ cm^-3^
Effective valence band density of state (nv300)	1.4 × 10^19^ cm^-3^
Effective Richardson constant of hole	100 A cm^-2^ K^-2^
Hole mobility in the surface region (µp)	100 cm^2^/V.s
Carriers (hole) lifetime (TAUP0)	1.0 × 10^–9^
Hole saturation velocity (VSATP)	1 × 10^7^ cm/s
Electron affinity EA	-1.3 eV

We investigated the drift–diffusion model, which is the simplified form of the charge transport sheet model in Atlas. The mechanism used in this work is interface fixed charge, which defines the space charge using Poisson’s equation with the ionized donor and acceptor. The mathematical model is established using the fundamental equations, including Poisson’s equation based on the Maxwell’s laws. Typically, the Poisson’s equation is formed based on the electrostatic potential $\varphi$ and space charge density $\rho$ as depicted as the following equation:

4 $$\nabla \left( {\varepsilon \nabla \varphi } \right) = - \rho$$

In general, the space charge contains the mobile and fixed charges (electron, hole, and ionization energy of impurities). We assumed three surface-charge models in this work, including the negative, positive, and neutral charge models of − 5 × 10^12^ cm^−2^, and 5 × 10^11^ cm^−2^, respectively. The continuity equation of electron and hole is given as:

5 $$\frac{\partial n}{{\partial t}} = \frac{1}{q}\nabla J_{n} + G_{n} - R_{n}$$

6 $$\frac{\partial p}{{\partial t}} = \frac{1}{q}\nabla J_{p} + G_{p} - R_{p}$$

where *n* is the electron concentration and *p* is the hole concentration, $J_{n}$ and $J_{p}$ are the electron and hole current densities, $G_{n}$ and $G_{p}$ are the electron and the hole generation rates. $R_{n}$ and $R_{p}$ are the recombination rates of electron and hole, respectively, and *q* is the magnitude of the charge on the electron. The carrier continuity equation in this model is then used for carrier density improvement as a result of transport, generation, and recombination processes for the specific hole, which only creates a wide bandgap of diamond and p-channel unipolar device by means of simulation.
